# Is the happiness of Chinese truly the highest in the world? The impact of basic public services on happiness

**DOI:** 10.3389/fpubh.2023.1271593

**Published:** 2023-10-30

**Authors:** Na Mu, Shaoting Li, Zhengbing Wang

**Affiliations:** College of Economics and Management, Northwest A&F University, Yangling, Shaanxi, China

**Keywords:** basic public services, happiness, inequality, social trust, ordered probit model

## Abstract

Based on the survey report by the United Nations Sustainable Development Solutions Network (SDSN) and Ipsos Group, the world ranking of Chinese people’s happiness shows a significant gap. This study attempts to analyze the subjective well-being of Chinese residents through public database from the China Household Finance Survey Center in 2017. An ordered Probit model is constructed to investigate the impact of non-monetary factors, specifically basic public services, on the subjective well-being of Chinese people. The results indicate that: (1) The subjective well-being of Chinese residents is found to be lower than what the survey report indicated. (2) Basic public services have a significant positive impact on residents’ happiness. (3) Social trust played a moderating role, positively influencing the relationship between basic public services and residents’ happiness. (4) The impact of basic public services on happiness varied significantly depending on factors such as age, registered residence, and places of residence. To enhance the happiness of Chinese residents, it is recommended to focus on improving the equalization of basic public services and establishing a robust basic public service system. These measures can effectively contribute to the overall well-being and happiness of the population.

## 1. Introduction

Happiness is a comprehensive evaluation of an individual’s quality of life and a subjective feeling. Enhancing residents’ happiness is a significant subject in the field of happiness economics (1). Earlier studies on happiness primarily concentrated on two areas: the determinants of happiness and its consequences. Firstly, regarding the determinants of happiness, scholars explored the influence of economic factors, such as macroeconomic conditions, assets, and income, on happiness (2, 3). However, the “Easterlin paradox” indicates a diminishing association between economic growth and residents’ happiness (4). Increasingly, researchers have turned their attention to non-monetary factors such as individual characteristics, natural environment, and social stability (5, 6). Political systems and government actions have also been identified as factors influencing residents’ happiness (7, 8), including democracy (9), corruption issues (7), urban scale (10), and environmental factors (11). Furthermore, the effects of happiness on individuals and families have been studied extensively. Increased happiness positively impacts employees’ organizational commitment and productivity (12, 13). Higher happiness levels lead to more favorable decision-making (14, 15). Additionally, happiness is associated with higher social and overall family consumption (16) and greater participation in commercial insurance (17). However, in the Dutch context, happiness has been linked to a reduced likelihood of investing in risky financial assets and obtaining insurance (18).

Research on basic public services, as a crucial institutional arrangement for ensuring and enhancing people’s well-being, has been extensively explored with a focus on equalization, policy effects, and performance evaluation. However, the issue of non-equalization of basic public services in China remains a prominent concern. Studies have revealed significant disparities in medical and health expenditure (16), and while the equalization level of public education has improved, regional imbalances persist (19). Commercialization of housing markets has further widened the gap in accessibility to public services between central cities and outer suburbs (20).

The importance of basic public services extends to poverty alleviation, rural revitalization, and promoting common prosperity (21). Nonetheless, some researchers have pointed out a decline in the equalization of basic public services in China, with transfer payments proving insufficient in promoting equalization (22). Assessing the performance of basic public services, scholars have observed regional disparities in efficiency across 30 Chinese provinces from 2006 to 2019, showing a pattern of “high in the east and low in the west” (23). Contrasting this, in Germany, property rights and organizational structures significantly impact public service provision, with public ownership proving more efficient than mixed or private ownership (24). Research has also emphasized the role of cooperation between cities in improving the efficiency of public service supply (25). Additionally, the development of e-government has demonstrated significant improvements in the net benefits of public services (26). Establishing performance monitoring and evaluation mechanisms for basic public services can help address inefficiencies and encourage citizen participation in co-producing public services (27).

The provision of basic public services represents a crucial policy domain characterized by the closest interaction between the government and citizens, and its influence on residents’ happiness has emerged as a prominent topic in the realm of happiness economics (28). Prior research has demonstrated the significance of various factors on residents’ happiness, including housing security (29), social support (30), public education (31), career decidedness (32), and disability care (33). The efficacy of government-provided basic public services bears a positive impact on citizens’ happiness. Enhancing the quantity, quality, and accessibility of these services serves as a pivotal approach to elevate residents’ happiness (34).

Numerous studies have addressed the factors influencing happiness and its consequential effects. However, certain aspects warrant further improvement. Firstly, existing research has predominantly focused on specific types of basic public services (e.g., public education, social insurance, healthcare, housing security), failing to comprehensively capture the holistic impact of such services. Secondly, there is a paucity of research investigating the relationship between satisfaction with basic public services and residents’ happiness, highlighting the need to explore the influence of basic public service satisfaction on overall well-being. To address these gaps, this study utilizes data from the 2017 China Family Finance Survey (CHFS2017) and integrates basic public service satisfaction into the happiness analysis framework. Empirical testing is conducted to assess the impact of basic public service satisfaction on residents’ happiness, with a further exploration of the moderating effect of social trust. The ultimate aim is to enhance residents’ happiness and well-being.

## 2. Hypothesis

### 2.1. The impact of satisfaction with basic public service on residents’ happiness

Basic public services play a crucial role in meeting residents’ essential needs for survival and development, directly influencing their overall well-being (35). Factors like medical care, older adult care, employment security, and housing have a direct and positive impact on residents’ happiness (24, 29, 36). When the basic public services in a region effectively meet residents’ needs, their satisfaction increases, leading to an enhancement in their quality of life and overall happiness. Conversely, dissatisfaction with local basic public services, such as prolonged air pollution issues (37), or inadequate medical insurance reimbursement and complex procedures (38), exposes residents to the risks of physical and mental health challenges, consequently reducing their sense of happiness.

The development of basic public services in China exhibits notable disparities, particularly evident in the urban–rural and regional dimensions. The enduring urban–rural dual structure perpetuates significant discrepancies in the provision of basic public services. Presently, urban areas enjoy ample and at times excessive access to these services, while impoverished regions, especially in public healthcare and social security, still require substantial improvements. Moreover, economic development plays a crucial role in shaping the supply pattern of basic public services, creating a distinct east-to-west ladder-shaped distribution with significant regional variations (23). The economically advanced eastern region benefits from substantial public finance investment and a robust financial system, resulting in a more robust provision of basic public services compared to less developed areas. Furthermore, it is essential to consider different demographic needs for basic public services. Various age groups exhibit distinct preferences and priorities. For instance, teenagers emphasize public education, young adults prioritize employment services, and the older adult place greater significance on social security and public healthcare. These distinct demands for public services have varying impacts on overall happiness levels, depending on the satisfaction level with the services. In light of these observations, the study proposes the following hypotheses:

*Hypothesis* 1: The level of residents’ happiness is positively influenced by their satisfaction with basic public services.

*Hypothesis* 2: The relationship between satisfaction with basic public services and residents’ happiness varies significantly based on age, household registration, and residential location.

### 2.2. Moderation effect of social trust

Social trust, as a crucial element of social capital, entails a subjective assessment of an individual’s level of trust, influenced by personal, communal, and social perceptual factors (39). Studies have shown that higher levels of trust in others are associated with an increased sense of happiness (40). While social trust positively impacts public service satisfaction, the conversion efficiencies may vary (41). The enhancement of social capital, including trust norms and social networks, contributes positively to the relationship between public service satisfaction and residents’ happiness (42). As a form of social capital, the degree of social trust may moderate the impact of basic public service satisfaction on residents’ happiness. Firstly, from a demand satisfaction perspective, individuals with higher social trust are more likely to avail social support from existing basic public services, fulfilling their survival and developmental needs, thereby enhancing their happiness. Secondly, in terms of resource acquisition, individuals with elevated levels of social trust are prone to achieving higher returns when exploring opportunities, leading to an improvement in personal and family welfare, albeit with potentially greater risks. Thirdly, from the perspective of physical and mental health, individuals with higher trust levels are more inclined to utilize existing public service resources in a relaxed state, thereby promoting their overall well-being and mental health. In conclusion, the following hypothesis is posited:

*Hypothesis* 3: Social trust plays a positive role in moderating the satisfaction of basic public services and residents’ happiness.

## 3. Materials and methods

### 3.1. Participants and procedure

The China Household Finance Survey (CHFS) is a comprehensive nationwide sampling survey project conducted by the China Household Finance Survey and Research Center at the Southwestern University of Finance and Economics. Commencing in 2011, the CHFS has undertaken biennial follow-up surveys, employing nationally representative samples. The survey questionnaire encompasses various aspects such as individuals and families, population and employment, assets and liabilities, insurance and security, expenditure and income, governance, and other relevant domains. The CHFS is esteemed for its high questionnaire response rate and data quality, making it a reputable and authoritative resource within the Chinese database landscape. Its extensive usage and recognition further attest to its credibility.

CHFS employed a rigorous sampling methodology for participant selection and conducted multiple rounds of tracking surveys using a household-based approach. The CHFS sampling design comprises two key components: the overall sampling scheme and the terminal sampling scheme.

Specifically, the overall sampling scheme of this project utilized a stratified, three-stage, and proportional-to-size (PPS) sampling design. To manage costs effectively, the initial CHFS survey round targeted a range of 8,000 to 8,500 households. The primary sampling units (PSUs) consisted of 2,585 cities/counties nationwide, excluding Tibet, Xinjiang, Inner Mongolia, Hong Kong, and Macau. PPS sampling was employed at each stage, with weights assigned based on the population (or number of households) of the sampling unit. In the first stage, the 2,585 cities/counties were stratified into 10 groups based on *per capita* GDP. Within each stratum, eight cities/counties were selected using PPS with population weights. In total, 80 cities/counties were selected in the first stage, covering 25 provinces across China. In the second stage, four neighborhood committees/village committees were directly sampled from each of the 80 selected cities/counties. The third stage determined the terminal sample size (i.e., the number of households sampled from each neighborhood committee/village committee) based on urban–rural distinctions and regional economic development levels, ranging from 20 to 50 households, with an average of approximately 25 households. China Household Finance Survey (CHFS) recruited surveyors from Chinese universities and research institutions to complete this multistage tracking survey.

For this study, we utilize the 2017 data from the CHFS database, primarily due to two key considerations. Firstly, the CHFS has successfully completed five rounds of follow-up research, demonstrating a large sample size, extensive coverage, and robust representativeness, thereby ensuring the reliability of drawn conclusions. In CHFS2017, a total of 355 counties or districts, 1,428 residents’ committees, and 40,011 families were selected from 29 provinces. Secondly, the CHFS data for 2017 provides the requisite variables necessary for constructing the proposed model, adequately fulfilling the data requirements of this study.

This study focuses exclusively on household heads’ questionnaires, as they play a crucial role as primary decision-makers in household affairs and daily activities. Happiness, being a positive emotional experience, is believed to have the potential to spread within families, implying that the positive emotions and well-being of household heads may influence other family members. We specifically target household heads aged 18 to 80 years old and exclude samples with a total household income less than zero. Additionally, we remove observations with missing data on relevant variables, resulting in 31,343 valid observations.

### 3.2. Measures

#### 3.2.1. Dependent variable

The dependent variable in this study is happiness. We operationalize residents’ happiness using a 5-point scale response to question A4011c “In general, do you feel happy now?” The response options on the scale are as follows: “very unhappy” = 1, “unhappy” = 2, “general” = 3, “happy” = 4, and “very happy” = 5.

#### 3.2.2. Independent variable

The independent variable in this empirical study is the satisfaction level with basic public services. These variable captures individuals’ subjective evaluations after utilizing public services. Unlike objective quantitative measures, the satisfaction level provides insights into the quantity and quality of local basic public services. Respondents rated their overall satisfaction with basic public services based on their personal experiences, using a scale from 0 (representing very dissatisfied) to 10 (representing very satisfied).

#### 3.2.3. Moderator variable

Social trust is selected as the moderator variable in this study, drawing from previous research (42). We employ individual trust as a proxy for social trust, measured by the question “How much do you trust people you do not know?” in the questionnaire. Respondents rate their level of trust on a 5-point scale, where “very distrust” = 1, “not very trust” = 2, “general” = 3, “trust” = 4, and “very trust” = 5.

#### 3.2.4. Control variables

The main control variables are as follows: (1) Individual characteristics, including gender, age, age squared, household registration type, subjective general health, education level, Internet usage, marriage, endowment insurance, and medical insurance. (2) Family characteristics, such as housing, number of cars owned, total household income, and total household debt. (3) Regional characteristics, classified into three regions: east, middle, and west^1^.

Table 1 presents the means, standard deviations, and maximum and minimum values of the variables used in this paper, along with descriptive statistics of the main variables. Among all respondents, 0.92% of the respondents reported feeling very unhappy, 3.62% felt unhappy, 24.77% expressed general, 49.36% reported being happy, and 21.33% described themselves as very happy. The mean happiness score is 3.866, with a standard deviation of 0.819, indicating a relatively high overall level of happiness among Chinese residents, but with room for further improvement. Moreover, the mean satisfaction with basic public services is 7.135, with a standard deviation of 2.157, also indicating a relatively high level of satisfaction.

**Table 1:** Description of variables.

Variables	Mean	Std Dev	Maximum	Minimum
Happiness (5-point scale, very unhappy = 1, very happy = 5)	3.866	0.819	5	1
Satis_score (10-point scale, very dissatisfied = 0, very satisfied = 10)	7.135	2.157	10	0
Gender (male = 1, female = 0)	0.800	0.400	1	0
Age1 (year)	49.959	14.013	80	18
Age100 (age^2^/100)	42.554	17.670	91	3
Residence (rural = 1, 0 otherwise)	0.507	0.500	1	0
Health (5-point scale, very unhealthy = 1, very healthy = 5)	3.423	1.003	5	1
Education (9-point scale, never attended school = 1, doctoral degree = 9)	3.516	1.682	9	1
Marriage (married = 1, 0 otherwise)	0.873	0.334	1	0
Internet (yes = 1, 0 otherwise)	0.494	0.500	1	0
Endow_insur (yes = 1, 0 otherwise)	0.830	0.375	1	0
Medi_insur (yes = 1, 0 otherwise)	0.938	0.241	1	0
Family_house (yes = 1, 0 otherwise)	0.853	0.354	1	0
Cars	0.310	0.542	5	0
Total_debt (unit: ten thousand yuan)	6.296	28.493	2024	0
Total_income (unit: ten thousand yuan)	10.287	20.341	849.78	0.01
East (east = 1, 0 otherwise)	0.503	0.500	1	0
Middle (middle = 1, 0 otherwise)	0.263	0.440	1	0
West (west = 1, 0 otherwise)	0.234	0.424	1	0

### 3.3. Model settings

#### 3.3.1. Ordered Probit model

This study employs the ordered Probit model to examine the association between public service satisfaction and residents’ happiness, treating happiness as a multi-classified ordered variable. The dependent variable, happiness, is defined on an integer scale from 1 to 5, representing levels of very unhappy, unhappy, general, happy, and very happy, respectively. The ordered Probit model is chosen as the preferred estimation method, as it provides a suitable probability framework for analyzing discrete selection problems in this context.

Suppose there is a latent variable happiness* that can represent the dependent variable *happiness* but cannot be observed,


(1) $$happiness_{i}^{*}=\alpha_{0}+\beta_{1}satis\_score+\beta_{2}X_{i}+\varepsilon_{i}$$

Where, $happiness_{i}^{*}$ is the latent variable of residents’ happiness, satis_score is the independent variable, *X_i_* is a series of control variables, *α_0_* is a constant term, *β_1_* and *β_2_* is the estimated parameter, and *ε_i_* is the random disturbance term.

Let $r_{1}<r_{2}<r_{3}<r_{4}$ be the unknown cutoff points, and define them as follows:


(2) $$happiness=\{\begin{array}{l} 1,ifhappiness^{*}\leq r_{1}\\ 2,ifr_{1}<happiness^{*}\leq r_{2}\\ 3,ifr_{2}<happiness^{*}\leq r_{3}\\ 4,ifr_{3}<happiness^{*}\leq r_{4}\\ 5,ifr_{4}<happiness^{*}\leq r_{5} \end{array}$$

Assumed random disturbance term *ε* follows the standard normal distribution, and use $\Phi \left(•\right)$ to represent the distribution function of the standard normal distribution. The conditional probabilities of *happiness* can be obtained as follows:


(3) $$\begin{array}{l} P\left(happiness=1|X\right)=P\left(happiness^{*}\leq r_{1}\right)=\Phi_{1}\\ P\left(happiness=2|X\right)=P\left(r_{1}<happiness^{*}\leq r_{2}\right)=\Phi_{2}\\ P\left(happiness=3|X\right)=P\left(r_{2}<happiness^{*}\leq r_{3}\right)=\Phi_{3}\\ P\left(happiness=4|X\right)=P\left(r_{3}<happiness^{*}\leq r_{4}\right)=\Phi_{4}\\ P\left(happiness=5|X\right)=P\left(r_{4}<happiness^{*}\leq r_{5}\right)=\Phi_{5} \end{array}$$

Where, $\Phi_{j}=\Phi \left(\varphi_{j}-f\left(X\right)\right)\left(j=1,2,3,4\right)$ is the probability density function. In the ordered probit model, the regression coefficients will be estimated using the maximum likelihood estimation method. As the model is nonlinear, the estimated coefficients do not directly represent the marginal effects of the parameters. Consequently, to better quantify their influence on the dependent variables, it becomes essential to calculate the marginal effects for each parameter.

#### 3.3.2. Moderation effect model

This study aims to explore the heterogeneity of social trust’s impact on residents’ happiness through a moderation effect model, formulated as follows:


(4) $$\begin{array}{l} happiness_{i}^{*}=a_{0}+\beta_{1}satis\_score+\beta_{2}satis\_score^{*}trust_{i}\\ \;\;\;\;\;\;\;\;\;\;\;\;\;\;\;\;\;\;\;\;\;\;\;\;\;\;\;\;\;\;\;\;\;+\beta_{3}trust_{i}+\beta_{4}X_{i}+\varepsilon_{i} \end{array}$$

To test the moderation effect of social trust, the study introduces the independent variables along with the interaction term $satis\_score^{*}trust_{i}$ representing social trust. All other variables remain consistent with the previous model. According to hypothesis 2, significant and positive coefficients (*β_1_* and *β_2_*) in model (2) would indicate a positive moderating effect of social trust on the relationship between public service satisfaction and happiness.

## 4. Results

### 4.1. Ordered Probit model regression results

Table 2 presents the results of models (1) to (3) with happiness as the dependent variable. Model (1) includes only the satisfaction with basic public services variable and controls for other factors. It reveals a statistically significant positive effect on residents’ happiness at the 1% significance level. In models (2) and (3), additional individual and family control variables are introduced incrementally. Despite these additions, the positive and significant impact of satisfaction with basic public services on residents’ happiness remains robust, aligning with hypothesis 1.

**Table 2:** The impact of satisfaction with basic public services on happiness.

Variables	(1) Ordered Probit	(2) Ordered Probit	(3) Ordered Probit
Satis_score	0.110^***^(0.003)	0.104^***^(0.003)	0.104^***^(0.003)
Gender		−0.022(0.017)	−0.026(0.017)
Age1		0.010^***^(0.001)	0.010^***^(0.001)
Age100		0.003^***^(0.000)	0.003^***^(0.000)
Residence		0.037^**^(0.015)	0.037^**^(0.015)
Health		0.227^***^(0.007)	0.221^***^(0.007)
Education		0.001(0.005)	−0.006(0.005)
Marriage		0.261^***^(0.020)	0.240^***^(0.020)
Internet		−0.015(0.015)	−0.031^**^(0.015)
Endow_insur		0.052^***^(0.018)	0.043^**^(0.018)
Medi_insur		0.078^***^(0.026)	0.069^***^(0.026)
Family_house			0.107^***^(0.018)
Cars			0.091^***^(0.013)
Total_debt			−0.001^***^(0.000)
Total_income			0.002^***^(0.000)
Middle			0.036^**^(0.015)
West			−0.014(0.016)
Cut1	−1.633^***^(0.029)	−0.025(0.060)	0.034(0.062)
Cut2	−0.952^***^(0.023)	0.682^***^(0.058)	0.742^***^(0.060)
Cut3	0.226^***^(0.021)	1.904^***^(0.058)	1.968^***^(0.060)
Cut4	1.604^***^(0.023)	3.328^***^(0.059)	3.395^***^(0.061)
*N*	31,343	31,343	31,343

(1) *, * * and * * * represent the significance level of 10, 5 and 1%, respectively. (2) Cut1−cut4 is the tangent estimate of the ordered Probit model. (3) The corresponding robustness standard error is shown in the brackets below the estimated value. The following table is the same.

Models (2) and (3) provide additional insights. As indicated in Table 1’s descriptive statistics, approximately 80% of household heads are male, while females constitute one-fifth of the sample. Researchers acknowledge that the wide data distribution might affect statistical significance. Nonetheless, the results of this study demonstrate that women’s sense of happiness tends to be higher than men’s, although the difference is not statistically significant. These findings are consistent with the outcomes reported by other scholars (43). The observed trend may be attributed to the greater social and economic responsibilities borne by males, leading to higher stress levels in comparison to females (44). Additionally, the use of the Internet has shown a negative correlation with happiness. This may be attributed to factors such as prolonged addiction to mobile apps and frequent online communication, which could potentially weaken family relationships (45). Furthermore, individuals browsing information on the Internet may experience heightened material and spiritual expectations, intensifying feelings of comparison, and occasionally leading to frustration. However, the impact of education level on residents’ happiness was found to be statistically insignificant. Yet, higher education levels may correspond to elevated expectations of education returns, potentially weakening overall happiness. Model (3), with the inclusion of family control variables, reaffirms the negative influence of gender and Internet use on happiness. Moreover, education level and family debt were observed to negatively affect happiness, with debt significantly reducing household heads’ happiness. Regression results of regional characteristic variables indicate a potentially negative impact on residents’ happiness when living in the western region, although the outcomes were not statistically significant. These findings suggest substantial disparities in the level of basic public services across different regions in China, emphasizing the imperative of promoting equal development in this domain as a significant challenge.

In the ordered Probit model, the regression coefficients primarily indicate the significance and direction of the impact, while our primary focus lies on the marginal utility of variables. Table 3 presents the marginal effects of control variables. The findings reveal that, when other control variables are at their mean levels, a standard deviation increase (2.157) in basic public service satisfaction can lead to a 0.2% reduction in the probability of residents feeling “very unhappy,” a 0.7% reduction in the probability of feeling “unhappy,” and a 2.4% reduction in the probability of feeling “general.” Additionally, it can result in a 0.5% increase in the probability of feeling “happy” and a 2.9% increase in the probability of feeling “very happy.” Overall, the regression results demonstrate that the satisfaction with basic public services significantly enhances residents’ happiness.

**Table 3:** Marginal utility analysis of happiness.

Variables	Coefficients	Very unhappy	Unhappy	General	Happy	Very happy
		(1)	(2)	(3)	(4)	(5)
Satis_score	0.104^***^	−0.002^***^	−0.007^***^	−0.024^***^	0.005^***^	0.029^***^
	(0.003)	(0.000)	(0.000)	(0.001)	(0.000)	(0.001)
Gender	−0.026	0.001	0.002	0.006	−0.001	−0.007
	(0.017)	(0.000)	(0.001)	(0.004)	(0.001)	(0.005)
Age1	0.010^***^	0.000^***^	−0.001^***^	−0.002^***^	0.000^***^	0.003^***^
	(0.001)	(0.000)	(0.000)	(0.000)	(0.000)	(0.000)
Age100	0.003^***^	0.000^***^	0.000^***^	−0.001^***^	0.000^***^	0.001^***^
	(0.000)	(0.000)	(0.000)	(0.000)	(0.000)	(0.000)
Residence	0.037^**^	−0.001^**^	−0.002^**^	−0.009^**^	0.002^**^	0.010^**^
	(0.015)	(0.000)	(0.001)	(0.003)	(0.001)	(0.004)
Health	0.221^***^	−0.005^***^	−0.015^***^	−0.052^***^	0.011^***^	0.061^***^
	(0.007)	(0.000)	(0.001)	(0.002)	(0.001)	(0.002)
Education	−0.006	0.000	0.000	0.001	0.000	−0.002
	(0.005)	(0.000)	(0.000)	(0.001)	(0.000)	(0.001)
Marriage	0.240^***^	−0.006^***^	−0.016^***^	−0.056^***^	0.012^***^	0.066^***^
	(0.020)	(0.001)	(0.001)	(0.005)	(0.001)	(0.005)
Internet	−0.031^**^	0.001^**^	0.002^**^	0.007^**^	−0.002^**^	−0.009^**^
	(0.015)	(0.000)	(0.001)	(0.004)	(0.001)	(0.004)
Endow_insur	0.043^**^	−0.001^**^	−0.003^**^	−0.010^**^	0.002^**^	0.012^**^
	(0.018)	(0.000)	(0.001)	(0.004)	(0.001)	(0.005)
Medi_insur	0.069^***^	−0.002^***^	−0.005^***^	−0.016^***^	0.003^***^	0.019^***^
	(0.026)	(0.001)	(0.002)	(0.006)	(0.001)	(0.007)
Family_house	0.107^***^	−0.003^***^	−0.007^***^	−0.025^***^	0.005^***^	0.029^***^
	(0.018)	(0.000)	(0.001)	(0.004)	(0.001)	(0.005)
Cars	0.091^***^	−0.002^***^	−0.006^***^	−0.021^***^	0.004^***^	0.025^***^
	(0.013)	(0.000)	(0.001)	(0.003)	(0.001)	(0.004)
Total_debt	−0.001^***^	0.000^***^	0.000^***^	0.000^***^	0.000^***^	0.000^***^
	(0.000)	(0.000)	(0.000)	(0.000)	(0.000)	(0.000)
Total_income	0.002^***^	0.000^***^	0.000^***^	0.000^***^	0.000^***^	0.000^***^
	(0.000)	(0.000)	(0.000)	(0.000)	(0.000)	(0.000)
Middle	0.036^**^	−0.001^**^	−0.002^**^	−0.008^**^	0.002^**^	0.010^**^
	(0.015)	(0.000)	(0.001)	(0.004)	(0.001)	(0.004)
West	−0.014	0.000	0.001	0.003	−0.001	−0.004
	(0.016)	(0.000)	(0.001)	(0.004)	(0.001)	(0.004)

*, ** and *** represent the significance level of 10, 5 and 1%, respectively.

Table 3 also show that ownership of houses and the resulting “wealth effect” contribute to reducing the probability of residents feeling “very unhappy,” “unhappy,” and “general” by 0.3, 0.7, and 2.5%, respectively, while increasing the probability of feeling “happy” and “very happy” by 0.5 and 2.9%. This highlights the significant positive impact of housing security on residents’ happiness. Moreover, good personal health positively influences happiness. Each unit of health improvement correlates with a decrease of 0.5, 1.5, and 5.2% in the probability of feeling “very unhappy,” “unhappy,” and “general,” and an increase of 1.1 and 6.1% in the probability of feeling “happy” and “very happy,” respectively. Furthermore, total household income and total debt exhibit a significant impact on happiness, but the regression coefficient is zero, indicating that these variables do not have a noticeable effect on residents’ happiness. This finding corroborates the existence of the “happiness paradox” in China.

### 4.2. Moderation effect test

Table 4 reports the results of the moderation effect test, which were improved by centralizing relevant variables to mitigate multicollinearity’s influence on the outcomes. The findings indicate a significant regression coefficient of 0.007 for the interaction term between satisfaction with basic public services and social trust, at the 1% statistical level. This signifies that social trust plays a positive moderating role in the relationship between basic public service satisfaction and residents’ happiness. Specifically, respondents with higher social trust experience a more pronounced impact of satisfaction with basic public services on their happiness compared to those with lower social trust. In essence, as social trust improves, the influence of satisfaction with basic public services on residents’ happiness becomes more pronounced. As a result, hypothesis 2 is confirmed.

**Table 4:** The moderation effect of social trust.

Variables	With interaction term	Without interaction term
Satis_score	0.105^***^(0.003)	0.106^***^(0.003)
Trust	−0.031^***^(0.007)	−0.032^***^(0.007)
Satis_score*trust		0.007^**^(0.003)
Controls	Yes	Yes
*N*	31,343	31,343

*, ** and *** represent the significance level of 10, 5 and 1%, respectively.

### 4.3. Robustness test

#### 4.3.1. Endogenous analysis

There may be endogenous problems caused by reverse causation and missing variables between residents’ satisfaction with public services and happiness. First, this paper aims to explore the impact of satisfaction with basic public services on happiness, but happiness may inversely affect residents’ satisfaction with public services, and residents with high happiness may have higher satisfaction with public services. Second, although this article controls as many variables as possible, there may still be a missing variable problem. Public service satisfaction and residents’ happiness may be influenced by other factors such as personal experience and psychological state. Endogenous problems arising from these two causes can lead to biased estimates.

When dealing with ordinal variables for both the dependent and independent variables, direct application of the instrumental variable method is not feasible. However, the Conditional Mixed Process (CMP) estimation method, combined with instrumental variables, offers a more effective approach to address the estimation bias resulting from endogenous issues (46). In this study, the CMP estimation method is utilized, employing terrain relief (Rdls) as the instrumental variable. Terrain relief satisfies the correlation condition since in areas with significant relief, residents may face challenges in accessing basic public services. Consequently, when the government provides more comprehensive and convenient services, residents’ satisfaction with basic public services may increase, thus meeting the correlation criteria for instrumental variables. Importantly, terrain relief does not directly impact residents’ happiness and satisfies the exogeneity requirement, making it a suitable instrumental variable for the analysis.

CMP involves the simultaneous estimation of two equations. The first equation estimates the effect of basic public service satisfaction on residents’ happiness, as represented by formula (1) above. The second equation, on the other hand, treats satisfaction with basic public services as the dependent variable and the instrumental variable as the independent variable. Table 5 reveals significant results in both stages of the CMP estimation. In the first stage, terrain relief positively influences satisfaction with basic public services at the 1% significance level, and the *F* value exceeds 10, indicating the absence of weak instrumental variables. In the second stage, employing terrain relief as the instrumental variable, the coefficient for satisfaction with basic public services remains significantly positive at the 1% level. This signifies that even after addressing the endogeneity of satisfaction with basic public services, its positive impact on residents’ happiness persists. A comparison with the marginal effect results from the ordered Probit model in Table 3 indicates that the CMP method yields larger absolute values for the estimated marginal effects when residents feel “unhappy,” “general,” and “very happy.” This suggests that the endogeneity of satisfaction with basic public services may have previously led to an underestimation of its impact on residents’ happiness. Overall, the estimation results based on the CMP method reaffirm the positive influence of satisfaction with basic public services on residents’ happiness, with relatively stable regression outcomes.

**Table 5:** CMP estimation of the impact of satisfaction with basic public services on happiness.

	First stage	Second stage	Marginal effect				
	Satis_score	Happiness	Very unhappy	Unhappy	General	Happy	Very happy
Satis_score		0.359^***^	−0.031	−0.024^***^	−0.047^***^	0.001	0.101^***^
		(0.108)	(0.030)	(0.005)	(0.009)	(0.006)	(0.032)
RDLS	0.043^***^						
	(0.013)						
Controls	Yes	Yes	Yes	Yes	Yes	Yes	Yes
F value (first stage)	11.28						
*N*	31,343	31,343	31,343	31,343	31,343	31,343	31,343

*, ** and *** represent the significance level of 10, 5 and 1%, respectively.

#### 4.3.2. Replace the dependent variable

To test the results’ robustness, we substitute the dependent variable with the indicator of whether the county provides more and higher levels of public services. Table 6 presents the regression outcomes for model (1), which utilizes the dependent variable alone, while model (2) and model (3) introduce personal and family characteristic independent variables incrementally. The results in Table 6 reaffirm the significant positive impact of increased provision of public services on residents’ happiness, with a significance level of 1%. These findings are consistent with the ordered Probit regression results, further confirming the robustness of the analysis.

**Table 6:** Robustness test: replace the dependent variable.

Variables	(1) Ordered Probit happiness	(2) Ordered Probit happiness	(3) Ordered Probit happiness
Highlevel_serv	0.247^***^(0.013)	0.234^***^(0.013)	0.234^***^(0.013)
Controls	Yes	Yes	Yes
*N*	31,343	31,343	31,343

*, ** and *** represent the significance level of 10, 5 and 1%, respectively.

#### 4.3.3. Change the regression method

The dependent variable is an ordered categorical variable, and the suitable estimation method is the ordinal Logit. Model (1), model (2), and model (3) present the regression results using independent variables alone and gradually introducing personal and family characteristics. Table 7 demonstrates that the satisfaction with basic public services has a significant positive impact on residents’ happiness after employing the ordinal Logit regression. Table 7 also presents the marginal effect of happiness estimated using the ordered logit method, which is broadly consistent with the earlier estimates. The findings reveal that a one-unit increase in satisfaction with basic public services corresponds to a reduction in the probability of residents feeling “very unhappy,” “unhappy,” and “general” by 0.2, 0.6, and 2.8%, respectively. Conversely, it leads to an increase in the probability of residents feeling “happy” and “very happy” by 0.6 and 3.0%.

**Table 7:** Robustness test: change the regression method.

Variables	(1) Ordered logit happiness	(2) Ordered logit happiness	(3) Ordered logit happiness	Coefficients	Very unhappy	Unhappy	General	Happy	Very happy
Satis_score	0.204^***^	0.190^***^	0.191^***^	0.191^***^	−0.002^***^	−0.006^***^	−0.028^***^	0.006^***^	0.030^***^
	(0.005)	(0.005)	(0.005)	(0.005)	(0.000)	(0.000)	(0.001)	(0.000)	(0.001)
Controls	Yes	Yes	Yes	Yes	Yes	Yes	Yes	Yes	Yes
*N*	31,343	31,343	31,343	31,343	31,343	31,343	31,343	31,343	31,343

*, ** and *** represent the significance level of 10, 5 and 1%, respectively.

### 4.4. Heterogeneity analysis

Based on the regression and robustness analyses conducted above, it is evident that higher satisfaction with basic public services effectively enhances happiness. However, significant disparities exist in development among different regions and between urban and rural areas in China, and residents’ happiness is influenced by diverse factors such as family income, physical condition, and working environment. To explore the impact of public services on the happiness of various demographic groups, this study examines the heterogeneity of three subsamples based on household registration type, regional differences, and age. The test results are presented in Table 8.

**Table 8:** Heterogeneity analysis.

Variables	Registered residence		Regions			Age					
	Rural	City	East	Middle	West	18–29	30–39	40–49	50–59	60–69	70–80
Satis_score	0.086^***^	0.131^***^	0.115^***^	0.092^***^	0.102^***^	0.131^***^	0.092^***^	0.097^***^	0.104^***^	0.110^***^	0.117^***^
	(0.004)	(0.005)	(0.004)	(0.005)	(0.006)	(0.010)	(0.008)	(0.006)	(0.006)	(0.007)	(0.011)
Controls	Yes	Yes	Yes	Yes	Yes	Yes	Yes	Yes	Yes	Yes	Yes
*N*	15,891	15,452	15,769	8,228	7,346	2,856	4,561	7,513	7,813	6,023	2,577

*, ** and *** represent the significance level of 10, 5 and 1%, respectively.

#### 4.4.1. Grouped by urban and rural areas

The impact of basic public service satisfaction on the happiness of rural and urban residents is significantly positive at the 1% level, but the “happiness effect” of urban residents is more significant. The possible reason is that basic public services such as medical care, education, and employment are more popular and perfect in urban areas, because residents tend to pursue more and higher-level public services. For example, some counties have special needs for swimming pools and gymnasiums. In terms of education, urban residents pay attention to famous teachers and advanced teaching equipment. In rural areas, however, limited by the level of cognition and economics, residents do not respond strongly to the quality of public services. The group regression results of other control variables show that the happiness of urban residents in using Internet is significant at the level of 1%, while that of rural residents is not significant.

#### 4.4.2. Grouped by region

The impact of satisfaction with basic public services on happiness is more pronounced in the eastern region (0.115), followed by the western region (0.102), while the central region exhibits the least impact (0.092). These findings indicate that residents in the eastern region experience a more significant “happiness effect” from basic public services compared to those in the central and western regions. The higher impact in the eastern region can be attributed to its relatively developed economy and extensive coverage of basic public services, leading to a higher level of satisfaction among residents. Conversely, the central and western regions face economic underdevelopment and imbalanced and inadequate supply of basic public services, resulting in weaker satisfaction and consequently a milder impact on residents’ happiness. The observed difference in happiness between the central and western regions underscores the objective existence of the “happiness paradox” phenomenon in China.

#### 4.4.3. Grouped by age

The impact of satisfaction with basic public services on the happiness of all age groups follows a positive U-shaped curve and is significantly positive at the 1% level, indicating that both the youth and the older adult experience higher levels of happiness compared to the middle-aged group. One possible explanation for this pattern is that middle-aged individuals typically encounter greater work and life pressures. Among the age groups, the youth aged 18–29 exhibit the strongest impact (0.131), likely due to this phase being the peak period of residents’ demand for public services. Access to convenient basic public services during this stage can effectively meet the diverse needs of the residents, contributing to higher happiness levels.

## 5. Discussion

This study employed the criterion of household registration location to examine the impact of public service satisfaction on the happiness of urban and rural residents. Empirical findings reveal that, both in urban and rural settings, public service satisfaction significantly and positively influences residents’ happiness. However, the positive effect on urban residents’ happiness is notably greater than that observed among their rural counterparts. Drawing from Maslow’s Hierarchy of Needs theory, it is observed that individuals tend to pursue higher-level needs only when their lower-level needs are satisfied. In the context of China, many urban areas exhibit a higher level of economic development and ample provision of public services. Consequently, residents in these prosperous urban regions shift their focus toward higher-level needs, such as indulging in high-quality public services, including museums, art exhibitions, concerts, and theatrical performances. Conversely, in rural China, the hierarchy of needs for farmers remains at lower levels, characterized by lower overall educational attainment, income levels, health conditions, and expression of needs, as compared to their urban counterparts. Hence, stimulation solely through improved public services is insufficient to elevate the happiness of rural residents. However, in recent years, with the implementation of the rural revitalization strategy, absolute poverty has been eradicated in rural China. Coupled with improvements in healthcare, living conditions, and other factors, the accessibility and availability of public services in rural areas have significantly improved.

In this study, respondents were grouped by age, with 2,856 samples in the 18–29 age group, 4,561 samples in the 30–39 age group, 7,513 samples in the 40–49 age group, 7,813 samples in the 50–59 age group, 6,023 samples in the 60–69 age group, and 2,577 samples in the 70–80 age group. Empirical results reveal that the influence of basic public service satisfaction on residents’ happiness is statistically significant at the 1% level across all age groups. Examining the impact within each age group, it is noteworthy that the effect of public services on individuals aged 18–29 is the most pronounced. This can be attributed to the fact that individuals in this age range are typically undergoing education, and an increase in educational attainment significantly affects residents’ happiness. Additionally, educational institutions provide a safe and comfortable environment that satisfies the social needs of respondents. The second highest impact is observed among respondents aged 70–80. Clearly, individuals in this age bracket are likely to prioritize public services related to healthcare, caregiving, and older adult care. China’s current urban and rural basic old-age insurance system and basic medical insurance system have partially alleviated the pressure on residents’ older adult care needs, especially in the context of a growing aging population trend.

The policy implications arising from the above findings are as follows: Firstly, prioritizing the promotion of equalization in basic public services is crucial for enhancing residents’ happiness. This necessitates the establishment of a fair, efficient, and sustainable system tailored to the actual needs of residents, with equalization as a fundamental starting point. Secondly, the government, as the primary provider of basic public services, should implement standardized performance evaluation and accountability mechanisms for public service supervision. Shifting from a “helmsman” role to that of a “servicer,” the government should reinforce the development of a service-oriented approach and steadily enhance residents’ political and social trust. Thirdly, policymakers should focus on resource allocation optimization and institutional improvement, offering targeted policy support for various basic public services based on differences between urban and rural areas, regions, and specific demands. Continual enhancement of supply efficiency, alongside the progressive realization of basic public service equalization, should be ensured.

## 6. Conclusion

This study utilizes CHFS2017 data to investigate the impact of satisfaction with basic public services on residents’ happiness. We analyzed the description statistical characteristics of the sample, established an ordered Probit model to explore the impact of public services satisfaction on residents’ happiness, and tested the mediating effect of social trust between satisfaction with basic public services and residents’ happiness. The empirical findings are as follows: (1) The assertion regarding Chinese people’s happiness ranking first globally is subject to doubt. (2) Satisfaction with basic public services significantly and positively affects residents’ happiness, a relationship that persists even after accounting for other control variables and addressing heterogeneity. To address endogeneity concerns in the benchmark regression model, terrain relief is employed as the instrumental variable, and CMP estimation corrects the endogeneity issue. The results consistently support the positive effect of basic public service satisfaction on residents’ happiness. (3) Social trust plays a moderating role by strengthening the positive impact of satisfaction with basic public services on residents’ happiness. (4) Sub-sample estimations reveal heterogeneous effects of basic public service satisfaction on residents’ happiness among different groups. Urban residents experience a more pronounced “happiness effect” from basic public service satisfaction compared to rural residents, and residents in the eastern region exhibit a more significant “happiness effect” compared to those in the central and western regions.

## Data availability statement

Publicly available datasets were analyzed in this study. This data can be found at: https://chfs.swufe.edu.cn/sjzx/sjsq.htm.

## Ethics statement

Ethical review and approval were not required for the study on human participants in accordance with the local legislation and institutional requirements. Written informed consent from the patients/ participants or patients/participants, legal guardian/next of kin was not required to participate in this study in accordance with the national legislation and the institutional requirements.

## Author contributions

NM: Conceptualization, Data curation, Methodology, Software, Visualization, Writing – original draft. SL: Data curation, Writing – original draft. ZW: Funding acquisition, Supervision, Writing–review & editing.

## Footnotes

^1^The eastern provinces of China include 11 provinces including Beijing, Tianjin, Hebei, Liaoning, Shanghai, Jiangsu, Zhejiang, Fujian, Shandong, Guangdong, and Hainan. Central provinces include Shanxi, Jilin, Heilongjiang, Anhui, Jiangxi, Henan, Hubei, and Hunan. Western provinces include Guangxi, Inner Mongolia, Chongqing, Sichuan, Guizhou, Yunnan, Shaanxi, Gansu, Ningxia, and Qinghai. The eastern region is used as a regional dummy variable.
